# Assessment of Surgical Strategies for Pilonidal Sinus Disease in the Netherlands

**DOI:** 10.7759/cureus.25050

**Published:** 2022-05-16

**Authors:** Eleonora A Huurman, Hidde A Galema, Christel de Raaff, Boudewijn Toorenvliet, Robert Smeenk

**Affiliations:** 1 Surgery, Erasmus Hospital, Rotterdam, NLD; 2 Surgery, Amsterdam UMC (University Medical Centers), Amsterdam, NLD; 3 General Surgery, Ikazia, Rotterdam, NLD; 4 General Surgery, Albert Schweitzer Ziekenhuis, Dordrecht, NLD

**Keywords:** flap techniques, excision techniques, minimal invasive techniques, pilonidal sinus disease, surgical procedures

## Abstract

Purpose

Pilonidal sinus disease (PSD) is a subcutaneous infection of the sacrococcygeal region due to entrapment of hair and/or debris. International guidelines recommend minimally invasive techniques and flap techniques. A Dutch guideline for the treatment of PSD is not available and this may lead to practice variation. The aim of this study was to perform a national survey on the surgical treatment of PSD in the Netherlands.

Method

An online survey was sent by e-mail to all surgeons and surgical residents of the Dutch Association for Surgeons. Respondents were asked to reflect on their preferences in the treatment of PSD, their perceived satisfaction with this treatment, and the need for national guidelines.

Results

A total of 819 (48.6%) of 1684 invitees responded to the survey, of whom 615 (37%) met the inclusion criteria. Traditional excision techniques were most frequently performed for all types of PSD (50.7%) followed by flap techniques (22.6%) and minimally invasive techniques (22%). Only 22.6% of the participants were satisfied with the current treatment and 82% supported the development of a national guideline.

Conclusion

Traditional excision techniques are most frequently performed for PSD in the Netherlands but the majority of surgeons and surgical residents are not satisfied with the current treatment. There is a demand for a national guideline.

## Introduction

Pilonidal sinus disease (PSD) is an infected tract under the skin in the gluteal cleft due to entrapment of hair and/or debris. It is a common disease for which surgery is performed in around 8000 patients per year in the Netherlands [1]. PSD is a condition mostly seen in patients between the age of 14 and 40 years [2]. Known risk factors for PSD are the male gender, an anatomically deep natal cleft, and a positive family history of PSD [3,4]. Recent studies have shown that occipital hair is regularly present in pilonidal nests [5]. A substantial proportion of patients with PSD refrain from work or study. Surgery is the main treatment but high recurrence rates and long wound healing times result in substantial costs for health care and society.

Many surgical treatments have been described in the literature. For decades, the standard surgical procedure for PSD has been excision of all infected tissue and surrounding subcutaneous fat and skin, resulting in an (often large) open wound in the natal cleft that is left to heal by secondary intention. Another treatment that is still used is primary closure in the midline after excision, but according to some national guidelines, this technique should be abandoned [6-8]. Other techniques currently gaining attention are minimally invasive procedures and primary wound closure techniques outside the midline such as the Bascom cleft lift and Karydakis flap. Although the promising results of these primary closure techniques have been published long ago, the standard surgical practice has not moved away from radical excision and secondary wound healing in the past 20 years.

Although PSD is a common disease, a national guideline for the management of this disease is currently lacking in the Netherlands. Since 2013, American, German, and Italian guidelines have been published [6-8]. These guidelines have in common that the type of treatment chosen depends on the severity of PSD. For example, according to the German guidelines, an asymptomatic PSD does not require treatment [6]. According to all three guidelines, minimally invasive techniques represent a promising treatment option for mild PSD and flap techniques for severe or recurrent PSD [6-8].

Despite these published guidelines, there remains a relative paucity of high-level evidence to define the best clinical practice of PSD. The aim of this study was to assess the current surgical management of PSD in the Netherlands. It was hypothesized that the surgical treatment of PSD is variable.

## Materials and methods

An online survey was conducted using SurveyMonkey (https://surveymonkey.com). The survey was developed by the principal investigators. The survey has been approved by the Medical Research Ethics Committees NedMec. The usability and technical functionality of the electronic questionnaire have been tested by the research team before fielding. The survey was sent by e-mail (closed survey) to all surgeons (1343) and surgical residents (341) of the Dutch Association for Surgeons. Their e-mail addresses were retrieved through the Dutch Association for Surgeons. The survey allowed for web-based entry. The participants were told the length of time of the survey (five minutes), who the investigators were, and the purpose of the study on the first page. No mechanisms were used to protect against unauthorized access. The survey was voluntary and no incentives were offered. The number of questionnaire items per page was one to three. Respondents were able to review and change their answers through a back button. The time frame of data collection was three weeks. Three reminders were sent to nonresponders. There was an automatic method for capturing responses. Cookies were used to assign a unique user identifier to each client computer. Duplicate entries were avoided by preventing users' access to the survey twice. Exclusion criteria were incomplete responses and responses by retired surgeons.

The following characteristics of the respondents were collected: position, surgical differentiation(s), and the number of individual PSD operations per year and per hospital.

Twelve questions were asked about the perioperative management of PSD. The options for the different surgical treatments were: (1) excision of the sinus with secondary wound healing, (2) excision of the sinus with primary closure in the midline, (3) Bascom cleft lift, (4) Karydakis, (5) Limberg/Dufourmentel, (6) excision of the sinus with primary closure outside the midline (not further specified), (7) pit picking, (8) pit picking with phenol, (9) pit picking with laser, (10) Bascom 1 (pit picking with lateral drainage), and (11) deroofing.

Surgical treatments were categorized into three groups: traditional excision techniques (1 and 2), flap techniques (3-6), and minimally invasive techniques (7-11).

Respondents were asked if they differentiated between mild and severe PSD or if they used a classification system. They were also asked to report on their perceived satisfaction with this treatment, their need for a national guideline, and their need for a universally accepted classification system guiding clinical practice.

Data were analyzed using IBM SPSS version 24.0 (IBM Corp., Armonk, NY). Descriptive statistics were used.

## Results

Study population

A total of 819 of 1684 invitees responded to the online survey (48.6% response rate). Of these responses, 178 were excluded due to an incomplete response and 26 because the respondents were not practicing anymore. In total, 615 participants were included for analysis. A total of 412 of 615 participants (67%) were surgeons and 203 (33%) were surgical residents. The characteristics of the respondents are shown in Table 1.

**Table 1:** Characteristics of the respondents

Study population	N (%), N = 615
Function	
Surgeon	412 (67)
Surgical resident	203 (33)
Practicing	
Netherlands	595 (96.7)
Abroad	20 (3.3)
Surgical differentiation (multiple answers possible)	
Gastrointestinal	278 (45.2)
Oncology	228 (37.1)
Trauma	108 (17.6)
Vascular	73 (11.9)
Pediatric	37 (6)
Lung	36 (5.9)
Number of individual PSD surgeries/year	
0-10	291 (47.3)
10-20	211 (34.3)
20-30	91 (14.8)
>30	22 (3.6)

Peri-operative assessment and management of PSD

The respondents classified the severity of PSD mostly on the presence of infectious signs or pus (60.8%), the presence of a lateral sinus cavity (46.7%), and the size of the sinus cavity (45.9%). Only 11 respondents (1.8%) used a classification system for PSD. The peri-operative assessment and management of PSD are shown in Table 2.

**Table 2:** The peri-operative assessment and management of PSD PSD: pilonidal sinus disease.

Peri-operative assessment and management of PSD	N (%), N = 615
Treatment of asymptomatic PSD	
Conservative	481 (78.2)
Surgical	134 (21.8)
Distinction in the severity of symptomatic PSD and adjusted treatment	
Yes	468 (76.1)
No	147 (23.9)
Severity of symptomatic PSD based on (multiple answers possible)	
Presence of infection or pus	374 (60.8)
Presence of lateral sinus cavity	287 (46.7)
Size of the sinus cavity	282 (45.9)
Complaints of patient	195 (31.7)
Number of midline pits	129 (21)
Classification system used	
No	604 (98.2)
Yes	11 (1.8)
Prophylaxis antibiotic	
Never	409 (66.5)
Sometimes	162 (26.3)
Always	44 (7.2)
Postoperative antibiotic	
Never	459 (74.6)
Sometimes	149 (24.2)
Always	7 (1.2)
Shaving advice after surgical treatment	
Yes	316 (51.4)
No	299 (48.6)
Epilation advise after successful wound healing	
Yes	467 (75.9)
No	148 (24.1)
Advice for permanent hair removal	
Laser	326 (53)
Patient choice	108 (17.6)
Depilatory cream	22 (3.6)
Other	11 (1.8)
No advise	148 (24)

Treatment of asymptomatic PSD

Approximately one-fifth of the respondents (21.8%) used surgical treatment for an asymptomatic PSD.

Treatment of all types of PSD

**Figure 1:**
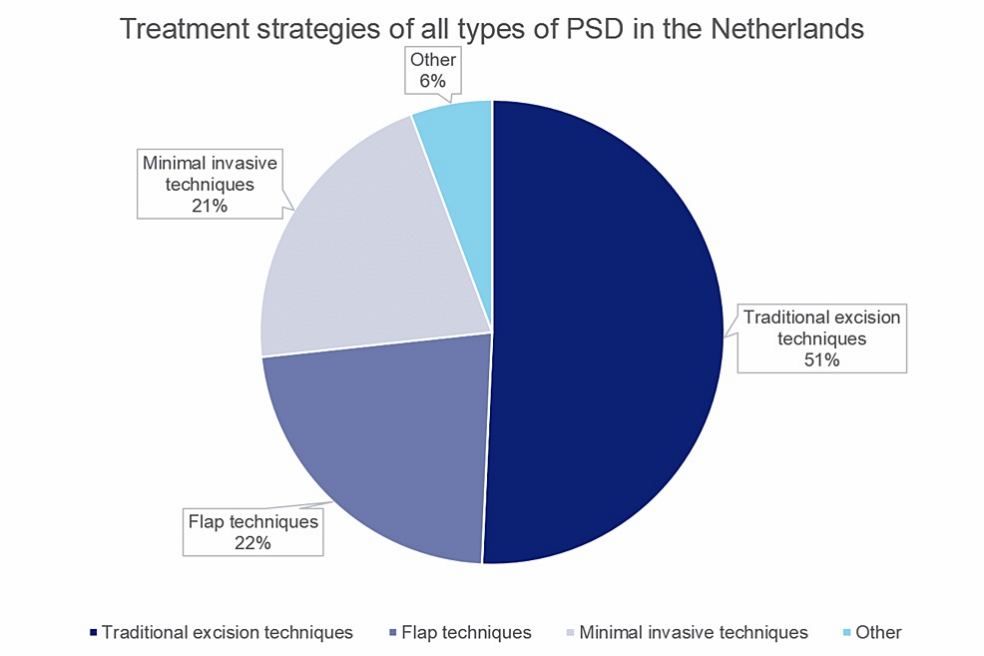
Surgical treatments in groups PSD: pilonidal sinus disease.

Traditional excision techniques were favored for all types of PSD by 50.7% of the participants. A total of 78.5% of this group performed excision with secondary wound healing. Flap techniques (22.6%) and minimally invasive techniques (22%) were used less frequently. At least four different types of minimally invasive techniques and three different types of flap techniques were performed. Treatment strategies for all types of PSD in the Netherlands are shown in Table 3. The surgical treatments in groups are shown in Figure 1.

**Table 3:** Treatment strategies for all types of PSD in the Netherlands PSD: pilonidal sinus disease.

Treatment strategies for all types of PSD in the Netherlands	N (%), N = 615
Secondary wound healing	245 (39.8)
Midline wound closure	67 (10.9)
Deroofing	33 (5.4)
Flap technique	139 (22.6)
Bascom cleft lift	28 (4.5)
Karydakis	12 (2.0)
Limberg/Dufourmentel	29 (4.7)
Unspecified	70 (11.4)
Minimally invasive treatment	96 (15.6)
Pit picking	5 (0.8)
Pit picking with phenolization	61 (9.9)
Pit picking with laser	17 (2.8)
Bascom 1	13 (2.1)
Other	35 (5.7)

Treatment of mild PSD

Excision of the sinus with secondary wound healing (25.9%) or primary closure in the midline (18.5%) as primary treatment for mild PSD was most frequently performed (44.4%). Minimally invasive techniques were performed less often (30.5%). Of these techniques, pit picking with phenolization was most frequently performed (67.9%). Only a minority of respondents (15.7%) used flap techniques as primary treatment for mild PSD. All treatment strategies for mild PSD in the Netherlands are shown in Table 4.

**Table 4:** Treatment strategies per type of PSD in the Netherlands PSD: pilonidal sinus disease; VAC: vacuum-assisted closure.

Treatment strategies per type of PSD in the Netherlands	N (%), N = 615		
	Mild, n (%)	Severe, n (%)	Recurrent, n (%)
Secondary wound healing	159 (25.9)	309 (50.2)	266 (43.3)
Midline wound closure	114 (18.5)	42 (6.8)	46 (7.5)
Deroofing	30 (4.9)	49 (8.0)	20 (3.3)
Excision of the sinus with VAC therapy	-	-	3 (0.5)
Flap technique	97 (15.7)	143 (23.3)	176 (28.6)
Bascom cleft lift	8 (1.3)	35 (5.7)	40 (6.5)
Karydakis	5 (0.8)	14 (2.3)	18 (2.9)
Limberg/Dufourmentel	2 (0.3)	30 (4.9)	54 (8.8)
Unspecified	82 (13.3)	64 (10.4)	64 (10.4)
Minimally invasive treatment	187 (30.5)	43 (7)	57 (9.3)
Pit picking	13 (2.1)	1 (0.2)	1 (0.2)
Pit picking with phenolization	127 (20.7)	23 (3.7)	34 (5.5)
Pit picking with laser	22 (3.6)	12 (2.0)	16 (2.6)
Bascom 1	25 (4.1)	7 (1.1)	6 (1.0)
Other	28 (4.6)	29 (4.7)	47 (7.6)

Treatment of severe PSD

The majority of respondents (50.2%) used secondary wound healing to treat severe PSD. Primary excision with midline closure for severe PSD was performed by 6.8% of the respondents. Only 7% of respondents used minimally invasive techniques. Flap techniques were performed by 23.3% of the respondents. All treatment strategies for severe PSD in the Netherlands are shown in Table 4.

Treatment of recurrent PSD

Excision of the sinus with secondary wound healing (43.4%) as primary treatment for recurrent PSD was performed most often. Minimally invasive techniques were performed less frequently (9.3%). Flap techniques were performed by 28.6% of the respondents. All treatment strategies for recurrent PSD in the Netherlands are shown in Table 4.

Satisfaction with the treatment of PSD

**Figure 2:**
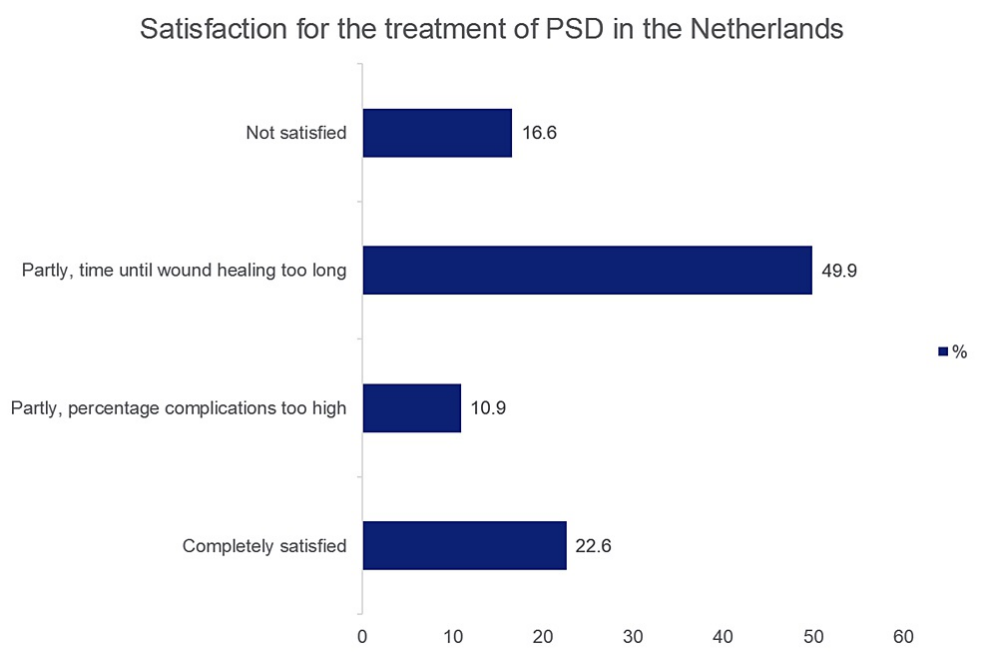
Satisfaction with the treatment of PSD PSD: pilonidal sinus disease.

Only a minority of the respondents (22.6%) were satisfied with the outcomes of their surgical care for PSD (Figure 2). The majority were not satisfied due to long wound healing times and high recurrence rates. More than 80% (82%) supported the development of a national guideline. More than 50% (52%) reported the need for a universally accepted classification system guiding clinical practice. The satisfaction with the treatment of PSD is shown in Figure 2.

## Discussion

Surgeons and surgical residents in the Netherlands perform a wide excision with secondary wound healing in the majority of patients with PSD, irrespective of the severity of the PSD. It seems curious that this treatment is still so popular amongst Dutch surgeons as these wounds often take months to heal. The wound healing times even increase with the increasing size of the defect, and the five-year recurrence rates are high (16-22%) [9,10]. Furthermore, excision with secondary wound healing is associated with sick leave, which could lead to considerable healthcare costs [11,12]. It is not surprising that during World War II, the surgeon general forbade this practice because it hospitalized 79,000 combatants for an average of 55 days [11].

Although open wound healing is still advocated as standard care by recent German and American guidelines, they also emphasize the problem of prolonged wound healing and the advantages of other techniques. Excision and primary closure are still being performed by 10.9% of respondents in the Netherlands. However, some international guidelines strongly advocate against this as there is a serious risk for wound breakdown resulting again in secondary wound healing or recurrence [6,11,12]. One could argue that excision with secondary wound healing should no longer be the preferred treatment and closure in the midline should not be performed at all.

The unsatisfactory outcomes of these outdated excision techniques have led to an ongoing search for alternatives. More promising procedures are the flap techniques that were introduced decades ago. Since the 1950s, various wound closure techniques that move the scar away from the depth and midline of the natal cleft have been described [9]. In contrast to traditional excision techniques, these procedures do not increase wound healing times with more extensive (complex) PSD. In addition, the five-year recurrence rates are much lower (2-10%) [9].

Despite these advantages, flap techniques are performed infrequently (22.6%) by Dutch surgeons and only 6.5% of respondents used a real off-midline flap technique (Bascom cleft lift or Karydakis). Half of the surgeons that did perform flap techniques did not specify their technique, and unfortunately, we could not ascertain the details for further analysis. An explanation for the limited use of flap techniques may be an unfamiliarity with the procedure and non-exposure to the technique during surgical training. Another explanation can be the more challenging learning curve for the flap techniques in comparison with “simple” excision and secondary wound healing.

To further optimize wound healing, reduce postoperative pain, and improve quality of life, innovative minimally invasive techniques were developed in the last decades. Deroofing, the Bascom I technique, and pit picking were introduced late in the 20th century, and have evolved with the addition of phenol, laser, or endoscopic techniques. These techniques have recently gained some popularity for (mild) PSD in the Netherlands and they are used by 22% of surgeons. There is a lot of practice variation but pit picking with phenolization was most frequently performed. The American guidelines advocate phenol application as an effective treatment that may result in rapid and durable healing [7]. However, the recurrence rate may be significantly higher than other techniques (20-25%) [9].

Minimally invasive procedures may be a good option compared to standard care, especially for simple, less complicated types of PSD that do not need wide excisions and/or flap techniques [6-8].

The severity of PSD may be the key to guiding treatment strategy and highlighting the importance of proper patient selection. The use of a comprehensive classification system that can differentiate between mild and severe diseases may be helpful [13].

There are eight classification systems reported in the literature but none is properly validated or practical enough for daily use in Dutch surgical practice [14]. Development and validation of such a classification system are needed, as, in our survey, half of the surgeons reported strong demand for this. Only 1.8% of the respondents used one of the non-validated classification systems that are available.

More than 75% of the respondents were not satisfied with the outcomes of their surgical care. The theory that most surgeons aim for better outcomes, but do not know which procedure to choose, was perhaps reflected in the fact that a large majority (82%) expressed the need for national practice guidelines. The development of Dutch national guidelines was initiated in early 2020 and will be published soon, but this may not lead to an immediate change in practice. The German guidelines advocate the use of flap techniques, but most German surgeons still perform excision with secondary wound healing [15]. A national implementation program for a standardized flap technique in the Netherlands may therefore help to introduce these procedures on a national level.

This study has some limitations that should be mentioned. First, the response rate of 48.6% was relatively low. We think, however, that it still offers a valid representation of Dutch practice as all Dutch surgeons were contacted, and not all of these surgeons perform surgery for PSD. Secondly, we used a non-validated questionnaire. Unfortunately, a validated questionnaire was not available in this field.

The surgical community in the Netherlands, and probably also abroad, knows too well that it is time to change practice for the (usually young) patients with PSD. The existing international guidelines and the upcoming Dutch guideline may be used to persuade surgeons in the Netherlands to change practice. Perhaps a united European effort for a European guideline would be even better.

## Conclusions

This nationwide survey showed that the surgical treatment of PSD in the Netherlands is variable, the majority of the surgeons are not satisfied with the outcomes of their surgical care for PSD, and surgeons express their wish for a national guideline on PSD. A national guideline is being developed. Traditional procedures, such as excision with secondary wound healing or primary closure in the midline, are still performed by the majority of Dutch surgeons, although this practice should probably be discontinued due to the unsatisfying outcomes. A variety of flap techniques and minimally invasive techniques are less frequently applied while these seem to be more promising regarding wound healing and should surely be implemented on a larger scale. No current consensus exists on treatment for PSD. High-level scientific evidence is much needed to improve health care for patients with PSD.
